# Mechanism of azole resistance in Candida glabrata isolates from India: clinical vs. induced perspectives

**DOI:** 10.1099/jmm.0.002083

**Published:** 2025-11-25

**Authors:** Kalpana Pawar, Ashutosh Singh

**Affiliations:** 1 Department of Microbiology, Medical Mycology Unit, Vallabhbhai Patel Chest Institute, University of Delhi, Delhi, India; 2 Department of Clinical Microbiology, School of Medicine, Trinity College Dublin, The University of Dublin, Dublin D08 RX0X, Ireland

**Keywords:** biofilm, *Candida glabrata*, chitosans, drug efflux pumps, *MSH2*, *PDR1*

## Abstract

**Introduction.** *Candida glabrata* is a pathogenic yeast in humans, recognized for its genomic plasticity and increasing prevalence of antifungal resistance, including multidrug-resistant phenotypes, especially in the US and European countries.

**Hypothesis.** This study hypothesizes that the resistance mechanisms in clinically resistant strains of *C. glabrata* differ from laboratory-generated resistant strains.

**Aim.** This study aims to understand the resistance mechanism in Indian clinical isolates of *C. glabrata.*

**Methodology.** A total of 240 clinical isolates of *C. glabrata* were tested for antifungal susceptibility and one resistant strain was artificially synthesized in the laboratory. Both clinical and lab-generated resistant strains were analysed for antifungal resistance using methods such as phenotypic assays, real-time quantitative PCR, Fluorescence-activated cell sorting (FACS) analysis and targeted gene sequencing. Mechanisms involving drug efflux pumps, mismatch repair pathways, ergosterol biosynthesis pathway and biofilm formation were systematically studied.

**Results.** Among clinical isolates, one susceptible-dose dependent strain and three fluconazole-resistant strains were identified. Both clinical and lab-generated resistant strains demonstrated antifungal resistance phenotypically, with increased expression of *CDR1*. Targeted gene sequencing revealed novel mutations in *PDR1*, while mutations in *MSH2* served as genotypic markers for resistance. Overexpression of *ERG11* was seen in a lab-generated resistant strain where a specific mutation was identified. Biofilm activity contributed to resistance in one of the clinical strains.

**Conclusion.** This study reports for the first time the fluconazole resistance mechanism in *C. glabrata* from India. The findings underscore the diversity of resistance mechanisms among clinical and lab-generated isolates, emphasizing the need for novel antifungal therapies to address these emerging resistance profiles effectively.

## Data Summary

All relevant data are within the manuscript and in the supplementary file. All gene sequences are deposited in the repository of National Center for Biotechnology Information (NCBI) GenBank, and accession numbers are allotted, which are added in the text.

## Introduction

Fungal infections remain a serious yet frequently underappreciated threat to global health. Current estimates suggest that they are responsible for 13 million infections and more than 1.5 million deaths globally [1]. While there is considerable uncertainty in measuring the burden of individual fungal diseases, evidence indicates that a relatively small number of them are responsible for most of this global impact. Among these, candidemia and invasive candidiasis stand out as major causes of fungal-related illness and death, second only to chronic pulmonary aspergillosis [2,3]

Traditionally, *Candida albicans* has been recognized as the most common cause of candidemia. However, in recent years, there has been a growing shift in the epidemiology of these infections, with an increasing number now caused by non-*albicans* species [4]. Of particular concern is *Candida glabrata*, which has become the second most frequently isolated species in bloodstream infections across several regions, including the USA, Canada, northwestern Europe and Australia, though it varies with geographical locations [5,9]. It is now responsible for up to one-third of all bloodstream infections caused by *Candida* species [10]. These infections are associated with high mortality rates, often ranging from 25 to 35%, and, in some cases, exceeding 50% in patients with hospital-acquired infections [11,15].

In response to the growing threat of fungal diseases, the World Health Organization published the first Fungal Priority Pathogen List in 2022. This list classified *C. albicans* and *Candida auris* as critical priority pathogens, while *C. glabrata*, along with *Candida tropicalis* and *Candida parapsilosis*, was placed in the high-priority group [2,3]. The distribution of *Candida* species responsible for candidemia varies by geography. While *C. glabrata* is dominant in North America, Northwestern Europe and Australia, species like *C. tropicalis* and *C. parapsilosis* are more frequently isolated in southern Europe, Asia and Latin America [13,16,18].

Although azole antifungals are still widely used to treat invasive *Candida* infections, particularly in settings with limited resources, current clinical guidelines recommend the use of echinocandins as the first choice for severe cases [19,22]. However, *C. glabrata* presents a particular challenge due to its natural low susceptibility to fluconazole and its ability to quickly develop resistance. Resistance rates vary by region, with fluconazole resistance reported in 10–40% of isolates in the USA and 1–22% in Europe [11]. Echinocandin resistance has also increased over time, with rates in *C. glabrata* rising from less than 2% to more than 12% in both Europe and the USA over the past decade [13,23].

The mechanisms underlying antifungal resistance in *C. glabrata* are diverse and reflect the organism’s remarkable ability to adapt to drug pressure. One of the central drivers of azole resistance is the overactivity of multidrug transporter proteins such as Cdr1, Pdh1 and Snq2 [12,14, 23,25]. This is typically caused by mutations in the transcription factor known as Pdr1, which enhances the expression of these efflux pumps and reduces intracellular drug accumulation [26,27]. Additionally, gain-of-function (GOF) mutations in genes like *UPC2A*, which regulates ergosterol biosynthesis, and Erg3, a key enzyme in the same pathway, contribute further to reduced susceptibility [28]. Mitochondrial dysfunction also plays a role by indirectly increasing the expression of resistance genes [29]. Resistance to echinocandins arises primarily from mutations in *FKS1* and *FKS2* which encode subunits of the enzyme beta-1,3-glucan synthase [5,12, 13]. These mutations reduce drug binding and enzymatic activity. Furthermore, activation of cellular stress responses, such as the calcineurin pathway and the enhancement of chitin content in the cell wall, can lead to increased tolerance [29]. Though less common, resistance to polyenes has been associated with mutations in ergosterol biosynthesis genes such as *ERG2* and *ERG6* [25]. Additional resistance mechanisms include chromosomal rearrangements that increase the copy number of resistance genes, defects in the mismatch repair gene *MSH2* and heteroresistance, where genetically similar cells within the same population show varying levels of drug susceptibility [7,13, 18]. Epigenetic changes, such as altered methylation and acetylation patterns in histone proteins, also influence the expression of key resistance genes, including *PDR1* and *ERG* [30]. Finally, stress response pathways, including the endoplasmic reticulum stress response and the cell wall integrity pathway involving Slt2, further support survival during antifungal treatment [31,32]. Together, these interconnected mechanisms make *C. glabrata* a particularly challenging pathogen to treat and highlight the urgent need for new therapeutic strategies.

In India, *C. glabrata* has traditionally been less prevalent and less resistant to antifungal agents. A 20-year surveillance study at a tertiary care centre in North India identified *C. glabrata* as the sixth most common cause of candidemia, following *C. tropicalis*, *C. albicans*, *Wickerhamomyces anomalus*, *Candida krusei* and *C. parapsilosis* [33]. Similarly, previous work by Singh *et al.* at the Medical Mycology Unit of the Vallabhbhai Patel Chest Institute found no resistance to either azoles or echinocandins among over 200 *C*. *glabrata* isolates collected from hospitals across northern and southern India. Notably, that study did detect mutations in the *MSH2* in phenotypically susceptible strains, suggesting a potential predisposition to resistance under antifungal selective pressure [34]. In contrast to earlier observations, a recent systematic review revealed that *C. glabrata* has become a significant non-*albicans Candida* (NAC) pathogen in India, representing 11.7% of clinical isolates and ranking third after *C. albicans* and *C. tropicalis*. Its prevalence is highest in western India (27%), and it shows worrying antifungal resistance, with overall rates of 20.7% among the highest in NAC species. Azole resistance is particularly notable, with fluconazole affected in over one-third of isolates, while regional surveillance reports resistance ranging from 10 to 38% to azoles and around 15% to amphotericin B [35]. Together with *C. albicans* and *C. tropicalis*, *C. glabrata* demonstrates high resistance across all regions. Notably, only about 35% of studies used broth microdilution (BMD), the Clinical and Laboratory Standards Institute (CLSI)-recommended gold standard, highlighting methodological variability in susceptibility testing [35].

Despite increasing concern over rising *C. glabrata* resistance, contemporary multicentre data from India remain limited. It remains unclear whether the historically low prevalence and high susceptibility of *C. glabrata* persist in the current therapeutic landscape. Moreover, there is scant information regarding the presence and distribution of resistance-associated genotypes such as mutations in *PDR1*, *UPC2A*, *ERG* pathway genes*, FKS1/2* hot spots and *MSH2* variants among current clinical isolates in India.

In the present study, we aimed to comprehensively investigate antifungal resistance in *C. glabrata* isolates collected from clinical centres across India. We analysed a new set of 240 isolates, examining both phenotypic resistance profiles and underlying molecular mechanisms. Additionally, to gain insight into resistance evolution, we generated a fluconazole-resistant strain *in vitro* by subjecting a susceptible isolate to stepwise drug exposure. Our findings provide valuable insights into the adaptive potential of *C. glabrata* under antifungal pressure and underscore the importance of continued surveillance and molecular characterization in regions where resistance has historically been infrequent.

## Methods

### Fungal isolates used in the study

The present investigation screened a total of 240 *C*. *glabrata* strains maintained in the fungal strain collection of the Medical Mycology Unit, Vallabhbhai Patel Chest Institute. These 240 strains were collected over a period of 5 years from 10 different clinical sources and preserved as glycerol stocks until use.

The isolates were recovered from a variety of clinical specimens. Out of 240 isolates, specimen information for 80 isolates could not be traced due to the retrospective nature of the study. Among the remaining isolates, the majority were urine samples (*n*=73), followed by blood samples (*n*=35), sputum (*n*=13), endotracheal secretions (*n*=10), bronchoalveolar lavage (*n*=3), tracheal tube secretions (*n*=3), high vaginal swabs (*n*=3), haemodialysis catheters (*n*=2) and a few from other sources, including hand swabs, catheters, stents, cerebrospinal fluid, body fluid, bone marrow transplant sample, vaginal swab and central venous pressure line.

All isolates were initially sub-cultured and confirmed on CHROM agar™ *Candida* medium for presumptive identification of * C. glabrata*. Final identification was performed using MALDI-TOF/MS (Bruker Daltonics, Germany) following the standard formic acid extraction protocol recommended by the manufacturer. Isolates yielding a score of ≥2.0 were categorized as *C. glabrata*.

### Antifungal susceptibility testing method

All 240 isolates of *C. glabrata* were subjected to antifungal susceptibility testing (AFST) by the CLSI–BMD method following method M27-A3 [34]. The isolates were tested against 10 antifungal drugs of three classes, i.e. azoles, echinocandins and amphotericin B. The antifungals tested were fluconazole (FLU) (Sigma, St. Louis, MO, USA), itraconazole (ITC) (Lee Pharma, Hyderabad, India), voriconazole (VRC) (Pfizer, Groton, CT, USA), posaconazole (POS) (Merck, Whitehouse Station, NJ, USA), isavuconazole (ISA) (Basilea Pharmaceutical, Basel, Switzerland), 5-flucytosine (5-FC) (Sigma), caspofungin (CFG) (Sigma), micafungin (MFG) (Sigma), anidulafungin (AFG) (Sigma) and amphotericin B (AMB) (Sigma). The drugs were tested for 10 (twofold) dilutions, and the drug concentration ranges were as follows: FLU, 0.25 to 128 mg l^−1^; ITC, VRC and AMB, 0.03 to 16 mg l^−1^; POS, ISA, AFG, MFG and CFG, 0.015 to 8 mg l^−1^; 5-FC, 0.125 to 64 mg l^−1^. *C. krusei* strain ATCC 6258 and *C. parapsilosis* strain ATCC 22019 were used as quality control strains [34,36].

### *In vitro* induction of fluconazole resistance

The clinically susceptible strain VPCI 431/P/21, isolated from sputum and exhibiting an fluconazole MIC of 16 mg l^−1^, was randomly selected for the induction of resistance. A single colony of this strain was inoculated into 10 ml of yeast extract peptone dextrose (YPD) broth and incubated at 37 °C for 16 h, after which 1% inoculum (50 µl into 5 ml) was serially transferred into fresh YPD broth (1:1000) supplemented with fluconazole at progressively increasing concentrations ranging from 0.5 to 128 mg l^−1^ (Fig. S1, available in the online Supplementary Material). A drug-free culture was maintained as a growth control during each passage, and following every transfer, 1 ml of culture was preserved by mixing with 0.5 ml of 50% glycerol and stored at −70 °C for subsequent antifungal susceptibility testing. At fluconazole concentrations ≥32 mg l^−1^, the MIC value increased to 64 mg l^−1^ and remained stable, even when cultured at 64 and 128 mg l^−1^ of fluconazole. To evaluate the stability of this resistance phenotype, strains serially exposed to 32, 64 and 128 mg l^−1^ were re-examined and consistently demonstrated an MIC of 64 mg l^−1^. After 30 days of serial exposure, a stable laboratory-derived fluconazole-resistant strain was established and designated as VPCI 9b and employed in subsequent experiments alongside its parental strain VPCI 431/P/21.

### Spot assay

To investigate the growth dynamics and fluconazole resistance response of clinical resistant strains, along with laboratory-induced strain VPCI 9b, a spot assay was performed on YPD agar plates containing twofold serial dilutions of fluconazole. Fluconazole-containing agar plates were prepared using a freshly prepared stock solution of fluconazole dissolved in DMSO, followed by serial dilutions ranging from 8.0 to 128 mg l^−1^. For culture preparation, 2% (100 µl) inoculum was taken from a 14- to 16-h grown primary culture (without antifungal supplementation) at 37 °C, inoculated into 5 ml of YPD medium and allowed to grow at 37 °C for 5 h. After 5 h, 0.1 OD_600nm_ cells corresponding to ~10⁶ cells ml^−1^ were taken from secondary culture in 0.85% saline, and ten-fold serial dilutions were done. Cell suspension of 5.0 µl was taken from each dilution of every strain and spotted on drug plates in addition to the control plate. Plates were left undisturbed for air drying under aseptic conditions and were incubated at 37 °C for 24 h, and their representative images were taken [37,39].

### E-test

All the above-mentioned fluconazole-susceptible, susceptible dose-dependent (SDD) and resistant clinical strains, along with the laboratory-generated fluconazole-resistant strain and the reference strain, were further confirmed for their fluconazole susceptibility profiles using the E-test. Testing was performed according to the manufacturer’s instructions (bioMérieux, Chicago, IL, USA), with modifications as recommended by the CLSI guidelines (M60, second ed., 2020) [40].

### Detection of mutations associated with azole resistance

All eight selected *C. glabrata* strains were screened by target sequencing for the detection of mutations in *ERG11*, *MSH2* and *PDR1* as described previously [36]. GenBank sequences with accession numbers KY967262, KY110696 and AY700584 were used as references for the *ERG11*, *MSH2* and *PDR1* sequences, respectively.

### Sterol extraction and ergosterol analysis

Sterol extraction was done using the 33% alcoholic KOH method [37], where organic content was silylated by adding 50 µl of BSTFA+TMCS (Supelco). Sterol analysis was done by GC–MS/MS (Shimadzu QP2010, Japan) instrument by running samples on a 30-m RESTEK column. Sterol identification was done by comparing their mass spectra with the NIST 17 library (National Institute of Standards and Technology, USA), and ergosterol analysis was done for each strain by absolute quantification.

### Rhodamine 6G extracellular efflux assay

This assay was done by using rhodamine 6G (R6G) (Sigma, USA) to find out the efflux activity of drug efflux pumps of all strains. Briefly, 2% inoculum taken from a 14- to 16-h grown primary culture (without any antifungal supplementation) was used to set up the secondary culture in 50 ml of YPD medium and incubated at 37 °C for 5 h. These cells were then pelleted, washed and finally resuspended as 2% cell suspension in PBS, where 5 mM deoxyglucose (Sigma, USA) was added to deenergize the cells. 10 µM R6G, which is a substrate for Cdr1p, was added to all tubes and kept on the rocker for 1 h. Cells were then pelleted and washed, and finally 2% cells were resuspended separately in PBS with glucose and PBS without glucose and again kept on the rocker. After an interval of 30 min, 1.0 ml of cells was taken from both sets (PBS with glucose and PBS without glucose) of the experiment and was pelleted at high speed. Supernatant was transferred to different tubes, which were used for measuring R6G efflux, and the pellet was finally resuspended in PBS buffer, which was then analysed by fluorescence-activated cell sorting (FACS) for R6G accumulation [41,42].

### Flow cytometry

FACS analysis was done to find out the R6G accumulation in fluconazole-resistant/susceptible strains using ~1 million cells, which were run on a BD FACS (Lyrics)™ flow cytometer. Analysis was done using BD FACS software v.1.5.

### Real-time quantitative PCR to confirm gene expression

To investigate the role of different genes involved in drug resistance, transcript level quantification was done by real-time polymerase using the ABI Prism 7500 Fast System (Applied Biosystems, CA, USA). First, RNA isolation was done from the logarithmic phase of secondary culture of all strains using Trizol Reagent. Here, 10 ml of late log phase cells was taken, pelleted and washed in DEPC-treated water. 1.0 ml of TRI reagent was added to the pellet, and lysis was done by vortexing three times (1 min vortex–1 min ice) using 0.5-mm glass beads. All proteins present in the lysate were precipitated by adding 200 µl of chloroform and keeping at room temperature for 15 min, which was removed by giving a high-speed spin, and total RNA was extracted by adding ethanol. DNAse treatment was given to the isolated RNA, which was quantified by the NanoDrop method, where the purity of the extracted RNA was checked to ensure an OD_260_/OD_280_ absorption ratio of >1.95. Subsequently, RNA was treated with RNase-free DNase I (NEB, MA, USA), and the purified RNA was used for the synthesis of cDNA using a High-Capacity cDNA Reverse Transcription Kit (Thermo Fisher) with random primers. Real-time PCR was performed using SYBR-Green (Applied Biosystems) by the 2-^ΔΔCt^ analysis method (where Ct is the threshold cycle) to determine the *n*-fold change in gene transcription. The single reaction mixture contained 1×Power SYBR Green PCR Master Mix, 3.0 µl of forward and reverse primers, 6.0 µl cDNA and RNase-free water at a final volume of 30 µl, which was aliquoted in duplicate at an equal volume of 15.0 µl. Samples were subjected to an initial holding stage at 50 °C for 20 s and at 95 °C for 10 min, followed by 35 cycles of 15 s at 95 °C and 1 min at 60 °C. Annealing and extension steps were undertaken at 60 °C, and fluorescence data were analysed with 7500 software v.2.0.6 (Applied Biosystems). *Cg ACT1* was used as a control for every experiment to normalize the Ct values. The data plotted were shown as averages with standard deviations of three independent experiments. Primer sequences were listed in Table S1 [43,47].

### Biofilm formation

Biofilm formation was studied, both in the absence and presence of serum using XTT solution, where first primary culture was set up in 10 ml of YPD media and was grown for 16 h at 37 °C. Secondary culture was set up by adding 2% (100 µl) of the primary culture to 5 ml of YPD media, which was kept for growth for 5 h at 37 °C. After 5 h, 100 µl of 0.5 OD_600nm_ cells diluted in 1× PBS were taken for each strain, including control (PBS only), and put in quadruplets in 96-well plate and kept at 37 °C for 90 min. After 90 min, unbound cells were removed, and bound cells were washed thrice with PBS, and 100 µl of RPMI media with 10% serum was added to each well and allowed to grow for 48 h at 37 °C. After 48 h, the media was removed, and 200 µl of XTT solution (1.0 mg ml^−1^ of XTT+0.4 mM menadione solution, HiMedia, in a ratio of 20:1) was added in the dark. The plate was covered with aluminium foil and kept at 37 °C for 2 h. After 2 h, 100 µl of cells was transferred to the next plate, and absorbance was taken at 495 nm. Absorbance of blank cells was subtracted from those containing cultures, and a graph was plotted as a ratio of reference culture to clinical culture [47,51].

### Scanning electron microscopy

To evaluate the morphological variation between the biofilms of various strains, scanning electron microscopy (SEM) was done as described. Briefly, 10 ml of the overnight grown cells was pelleted and washed thrice with 1× PBS. Primary fixation was done by keeping the cells in 2.5% glutaraldehyde (Sigma-Aldrich) in PBS (pH 6, Sigma-Aldrich) at 4 °C for 16 h. After primary fixation, cells were dehydrated by washing in successive concentrated alcohol (50%, 70%, 90% and 95%) at high r.p.m. for 2.0 min, and, finally, resuspended cells were mounted on coverslips and coated with gold under spatter coater and viewed under JEOL microscope JSM 6610L at 30 kV with ×5000 magnification [47,52].

### Transmission electron microscopy

Ten millilitres of overnight-grown cells in YPD media were pelleted and washed thrice with 1× PBS. Primary fixation was done by overnight keeping the cells in 2.5% glutaraldehyde in 1× PBS at 4 °C and was given to the analysis centre for further preparation, where secondary fixation was done in osmium tetroxide (OsO_4_) to enhance staining of intracellular membranes. After dehydration with successively more concentrated acetone (50%, 70%, 90% and 95%), clearing of the sample was done in toluene, and infiltration was done in different ratios of toluene and araldehyde (3:1, 2:2 and 3:2) for 2 h each, and the sample was embedded in araldehyde for polymerization. Ultrathin sections of 70–80 nm were cut using an ultramicrotome and stained with uranyl acetate and lead citrate. Samples were then viewed under a transmission electron microscope JEOL JEM-2100F at 120 kV [53,54].

### Statistical significance of data

As in this study, multiple groups (more than two) were compared; therefore, a single-factor ANOVA test was done to find the significant differences between means of different groups and to accept or reject the null hypothesis for different experiments, followed by a post hoc test, Tukey (honestly significant difference), to find out which groups were significantly different from each other. The confidence of interval was calculated at 95% to evaluate the precision or variability of the data. A student’s two-tailed t-test was done to find out the significance of the data by calculating the *P* value sequentially for each experimental group (strain) with respect to the control sample. Here, NS represents non-significant data which occur when observed *P* value ≥0.05, and * represents statistically significant data, and depending on the *P* value, * are assigned, i.e. * represents data ≤0.05, ** represents data ≤0.005 and *** represents data ≤0.0005.

## Results

### *In vitro* susceptibility profile of *C. glabrata* isolates

On testing the *in vitro* susceptibility profile of 240 isolates by AFST method, a high geometric mean (GM) value of 1.103 mg l^−1^ of fluconazole was found, where three isolates (1.25%) exhibited high MIC value of 64.0 mg l^−1^ and were categorized as resistant, while one isolate (0.41%) showed MIC value of 32.0 mg l^−1^ and was categorized as SDD. Remaining isolates (98.33%) were showing MIC values less than 32.0 mg l^−1^ and were categorized as fluconazole sensitive. Additionally, low GM values were found for other antifungal drugs, which is shown in Table 1.

**Table 1.** The MIC distribution of *C. glabrata* isolates against ten antifungal drugs – CLSI–BMD method

	No. of isolates with MIC (mg l^−1^) of																	
Drug	≤0.015	0.030	0.06	0.125	0.25	0.5	1.0	2.0	4.0	8.0	16.0	32.0	64.0	128				
**Echinocandin**															**MIC range**	**GM**	**MIC_50_**	**MIC_90_**
MFG	216	21	3	-	-	-	-	-	-	-	-	-	-	-	≤0.015–0.06	0.020	0.015	0.03
AFG	53	10	116	49	12	-	-	-	-	-	-	-	-	-	≤0.015–0.25	0.064	0.06	0.125
CAS	2	1	3	18	98	13	70	35	-	-	-	-	-	-	≤0.015–2	0.475	0.25	2
**Azoles, AMB and FC**																		
ITC	-	114	11	29	34	23	21	7	1	-	-	-	-	-	<0.03–4.0	0.130	0.125	1.0
VRC	-	178	38	12	6	6	-	-	-	-	-	-	-	-	<0.03–0.5	0.057	0.06	0.2
ISA	149	23	25	15	18	7	3	-	-	-	-	-	-	-	≤0.015–1	0.055	0.06	0.25
POSA	125	24	14	8	30	24	15	-	-	-	-	-	-	-	≤0.015–1	0.090	0.06	0.5
FLU	-	-	-	-	37	57	66	33	27	11	5	1	3	-	0.25–64	1.103	1.0	4.0
AMB	-	15	6	29	51	75	47	17	-	-	-	-	-	-	≤0.03–2	0.417	0.5	1.0
5-FC	-	-	-	240	-	-	-	-	-	-	-	-	-	-	≤0.125	0.157	0.125	0.25

A total of 240 isolates, including one reference strain ATCC 90030, were tested against all categories of antifungal drugs. Clinical break points of CAS and AFG are ≤0.12 mg l−1 (susceptible), 0.25 mg l−1 (intermediate) ≥0.5 mg l−1 (resistant), while for MFG are ≤0.06 mg l−1 (susceptible), 0.12 mg l−1 (intermediate) and ≥0.25 mg l−1 (resistant), respectively. Similarly, for FLU, it is ≤0.32 mg l−1 (susceptible dose dependent) and ≥64.0 mg l−1 (resistant), respectively. The table also shows the range of MIC for different drugs, their geometric mean (GM), MIC50 and MIC90, where 50 and 90% inhibition was observed.

#### Strains used

This study was done to understand the mechanism of azole resistance in Indian isolates of *C. glabrata.* As in this study, among 240 isolates screened only three fluconazole-resistant and one SDD strain were found. Therefore, for comparison, further study was restricted to eight strains of different susceptibilities, which included one reference strain ATCC 90030 (MIC 8.0 mg l^−1^, S), six clinical strains VPCI 226/P/20 (blood; MIC 64.0 mg l^−1^, R), VPCI 635/P/20 (blood; MIC 64.0 mg l^−1^, R), VPCI 250/P/21 (not known; MIC 32.0 mg l^−1^, SDD), VPCI 431/P/21 (sputum; MIC 16.0 mg l^−1^, S), VPCI 944/P/21 (sputum; MIC 8.0 mg l^−1^, S), VPCI 201/p/22 (blood; MIC 64.0 mg l^−1^, R) and one lab-generated or *in vitro* synthesized fluconazole-resistant strain VPCI 9b (MIC 64.0 mg l^−1^, R).

### Phenotypic study in the presence of fluconazole

To cross-check and validate the drug susceptibility of different strains used in this study, various phenotypic studies were done.

#### Spot assay

**Fig. 1.**
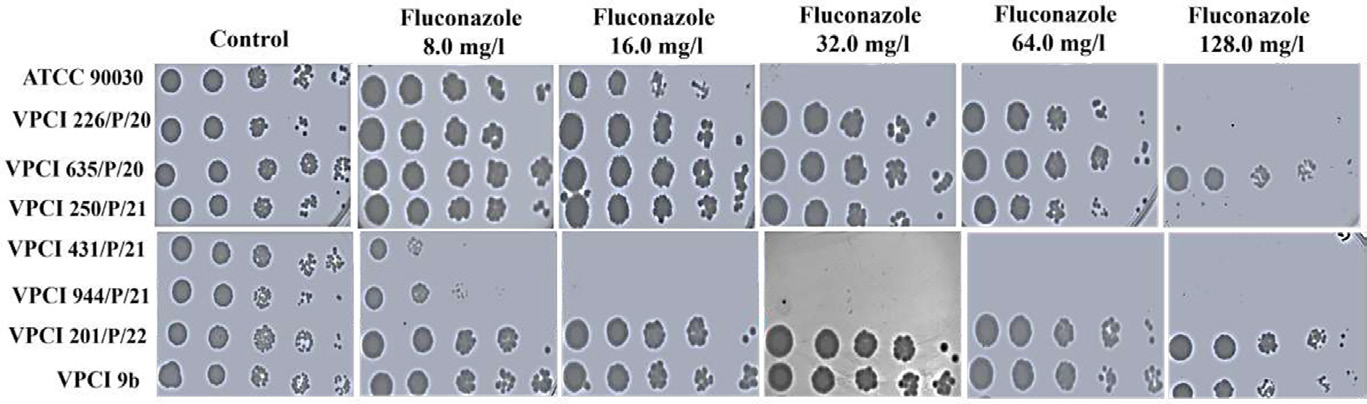
Effect of increasing concentrations of fluconazole on clinical and lab-generated strains of *C. glabrata*: the figure shows the effect on drug susceptibility of increasing concentration of fluconazole on different strains of *C. glabrata*. Cell suspension (5 µl) of serially tenfold diluted cells of log phase corresponding to cell numbers 1×10^7^, 1×10^6^, 1×10^5^, 1×10^4^ and 1×10^3^ were spotted from left to right in the same row. Spotting was done on a YPD agar control plate and fluconazole plates of five different concentrations, respectively. Plates were incubated at 37 °C for 24 h, and representative images are shown. The experiment was done twice.

In spot assay, it was found that in comparison to ATCC 90030, VPCI 431/P/21 and VPCI 944/P/21, strains like VPCI 226/P/20, VPCI 635/P/20 and VPCI 201/P/22 were growing till resistant values (64.0 mg l^−1^ and 128.0 mg l^−1^) of fluconazole in solid medium, while VPCI 250/P/21 was showing growth between susceptible and resistant strains (Fig. 1).

In a similar manner, VPCI 9b was also showing growth till resistant (64.0 mg l^−1^ and 128.0 mg l^−1^) values of fluconazole compared to both ATCC 90030 and VPCI 431/P/21, respectively, which was comparable to its MIC value (Fig. 1).

This experiment was done twice for all strains, with similar results found.

#### E-strip assay

This assay was done using fluconazole drug strips where formation of a clear inhibitory zone around the values on the strip confirmed their drug susceptibility. All results shown were comparable to their MIC values (Fig. S2).

### Role of drug efflux pumps in altering azole susceptibility in *C. glabrata*

#### Overexpression of *CDR1* leads to azole resistance

As is already known in *C. glabrata,* drug efflux pumps are the major player in azole resistance; therefore, their expression was first checked by real-time PCR, and their significance was tested by a one-way ANOVA test (descriptive statistic is shown in Table 2).

**Table 2.** The descriptive statistics of the ANOVA test. If the calculated *F* value is greater than the critical value and the *P* value is less than 0.05, then the null hypothesis is rejected. If the calculated *F* value is less than the critical *F* value and the *P* value is greater than 0.05, then the null hypothesis is accepted

Experiment	Calculated F value	*P* value	Critical *F* value	Interpretation
*CDR1* expression	78.02492091	3.59E−11<0.05	2.657197	Null hypothesis is rejected
*CDR2* expression	13.12640588	1.46E−05<0.05	2.657197	Null hypothesis is rejected
*PDR1* expression	0.752715	0.63309>0.05	2.657197	Null hypothesis is accepted
*ERG11* expression	1.466731	0.247706>0.05	2.657197	Null hypothesis accepted
Ergosterol level	0.70544	0.670661>0.05	3.500463855	Null hypothesis accepted
R6G influx in the absence of glucose	0.816299	0.59905>0.05	3.500464	Null hypothesis accepted
R6G influx in the presence of glucose	1.1507	0.419862>0.05	3.500464	Null hypothesis accepted
R6G efflux in the absence of glucose	20.76454483	0.000156<0.05	3.500464	Null hypothesis rejected
R6G efflux in the presence of glucose	2.530383	0.108419>0.05	3.500464	Null hypothesis accepted
Biofilm formation in the absence of serum	21.70314935	0.000132674<0.05	3.500464	Null hypothesis rejected
Biofilm formation in the presence of serum	27.41164089	5.55034E−05>0.05	3.500463855	Null hypothesis rejected

**Fig. 2.**
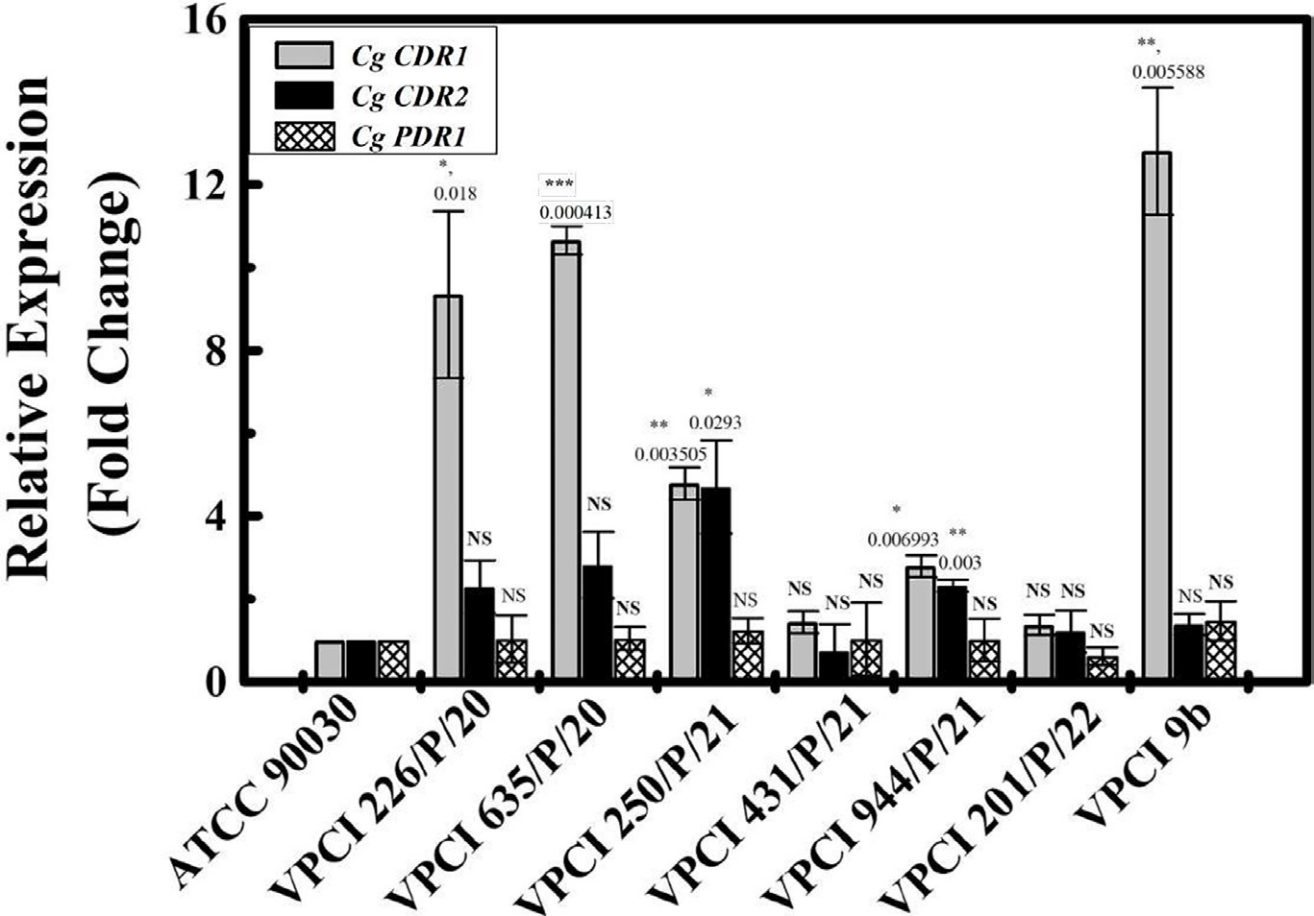
Azole resistance results in alterations in expression of drug efflux pump: The figure shows the transcript levels of *CDR1, CDR2* and *PDR1* in different strains of *C. glabrata* using ATCC 90030 as a control. Transcript levels are shown as fold change with sd. Confidence of interval (both minimum and maximum) at 95% is given in brackets with each value. *P* value is calculated for all compared to ATCC 90030. It was found that, compared to the reference strain ATCC 90030, overexpression of *CDR1* was seen in all other strains. A significant overexpression was seen in VPCI 226/P/20 (9.34684±2.012137, CI 4.35, 14.35), VPCI 635/P/20 (10.65774±0.340122, CI 9.81, 11.50), VPCI 250/P/21 (4.789559±0.38963, CI 3.81, 5.75), VPCI 944/P/21 (2.793841±0.261194, CI 2.14, 3.44) and VPCI 9b (12.80769±1.533073, CI 8.99, 16.6), but a non-significant overexpression was seen in VPCI 431/P/21 (1.434845±0.266365, CI 0.77, 2.09) and VPCI 201/P/22 (1.376057±0.245853, CI 0.76, 1.99), respectively. Similarly, transcript levels of *CDR2* and *PDR1* were also checked. In comparison to ATCC 90030, *CDR2* was significantly overexpressed in VPCI 250/P/21 (4.703971±1.123563, CI 1.91, 7.49) and VPCI 944/P/21 (2.319087±0.144055, CI 1.96, 2.67) only, but non-significant overexpression was seen in VPCI 226/P/20 (2.289907±0.63361, CI 0.71, 3.86), VPCI 635/P/20 (2.819312±0.795991, CI 0.84, 4.79), VPCI 431/P/21 (0.73637±0.650033, CI −0.87, 2.35), VPCI 201/P/22 (1.212697±0.509588, CI −0.053, 2.47) and VPCI 9b (1.388657±0.252489, CI −0.76, 2.01), respectively. In comparison to ATCC 90030, *PDR1* showed very little non-significant overexpression in all strains. In VPCI 226/P/20, transcript levels were 1.028255±0.569881 (CI −0.38, 2.44); in VPCI 635/P/20, 1.041234±0.282138 (CI 0.34, 1.74); in VPCI 250/P/21, 1.235125±0.300975 (CI 0.48, 1.98); in VPCI 431/P/21, 1.027561±0.87772 (CI −1.15, 3.20); in VPCI 944/P/21, 1.007419±0.516783 (CI −0.27, 2.29); in VPCI 201/P/22, 0.619856±0.212106 (CI 0.09, 1.14); and in VPCI 9b, 1.464108±0.476143 (CI 0.28, 2.64), respectively. These experiments were done thrice.

On checking the expression of *CDR1*, it was found that compared to ATCC 90030, although all clinical strains, irrespective of their MIC and drug phenotype, overexpressed *CDR1*, in comparison to VPCI 431/P/21 and VPCI 944/P/21, expression was high in VPCI 226/P/20 and VPCI 635/P/20, respectively. On the other hand, VPCI 201/P/22, being resistant, did not show any *CDR1* overexpression, and in VPCI 250/P/21, this expression lay between drug-sensitive and resistant strains (Fig. 2, Table 2). Similarly, the expression of *CDR2* was also checked, but very little or no expression was seen. In *PDR1*, no overexpression was seen irrespective of drug phenotype (Fig. 2, Table 2).

Similarly, in VPCI 9b, *CDR1* was found to be sufficiently overexpressed compared to both ATCC 90030 and VPCI 431/P/21. In fact, among all resistant strains, maximum overexpression was seen in this strain only. *CDR2* and *PDR1* were not overexpressed at all, as seen in other strains (Fig. 2, Table 2).

#### R6G assay

**Fig. 3.**
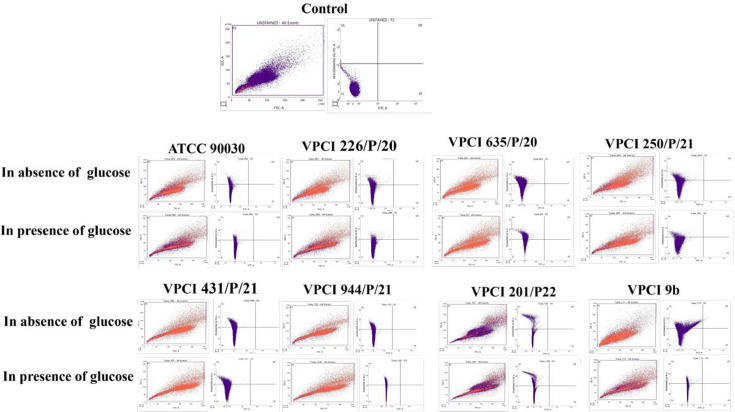
Flow cytometry analysis showing difference in R6G accumulation: the figure shows the accumulation of R6G both in the presence and absence of glucose using flow cytometry. For every strain representative, FACS images showing the total number of events and the number of events showing drug accumulation by quadrant analysis using gating, which was done with respect to the negative control, are shown in the figure. Different strains show variation in R6G accumulation both in the absence and presence of glucose. Every image in the figure shows different strains used in the study in both conditions. This experiment was done twice.

This assay was done to monitor the drug efflux activity of Cdr1p by both R6G accumulation (influx) and R6G efflux in all strains used in the study after 30 min of adding glucose. R6G accumulation was studied by FACS, while R6G efflux was studied by efflux assay, where the amount of R6G effluxed was studied. This experiment was done both in the absence and presence of glucose, and it was found that in the presence of glucose, VPCI 226/P/20 and VPCI 635/P/20 showed less R6G accumulation and more efflux, which was higher than ATCC 90030. On the other hand, VPCI 431/P/21 and VPCI 944/P/21 showed more R6G accumulation and less efflux (Figs3  4a, b and Table 3). In VPCI 250/P/21, both R6G accumulation and R6G efflux lay between sensitive and resistant strains, but in VPCI 201/P/22, no difference was seen in drug efflux both in the absence and presence of glucose. In fact, contrary to other resistant strains, more drug accumulation was seen in the presence of glucose. This effect was also seen in real-time PCR, where no change in *CDR1* expression was seen, depicting that in this resistant strain, drug efflux pumps were not active (Figs24a, b and Table 3).

**Fig. 4.**
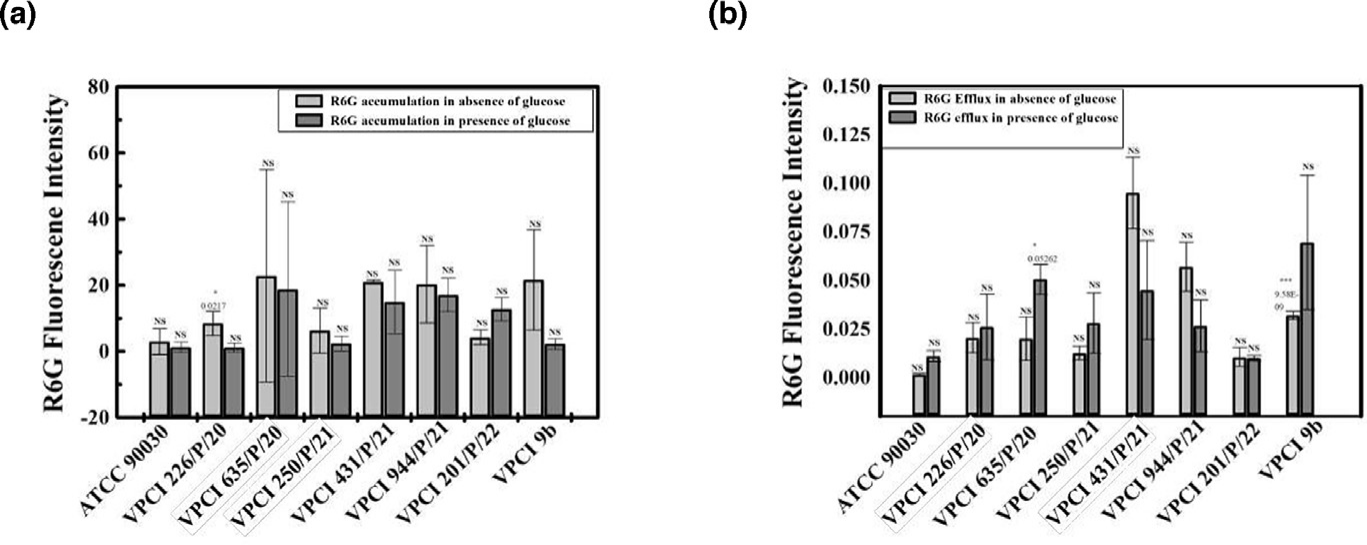
Analysis of drug efflux pumps by monitoring the R6G accumulation (influx) and efflux in the absence and presence of glucose: panel (a) shows the graph of R6G accumulation both in the absence and presence of glucose. Every bar is plotted as the mean of the number of events showing fluorescence±sd of two independent experiments, and the CI value (both minimum and maximum) at 95% is also given. *P* value is calculated with respect to ATCC 90030, which is shown above the bar. In the absence of glucose, a non-significant alteration in R6G fluorescence intensity was seen in all strains except VPCI 226/P/20. Number of events showing R6G fluorescence was 2.95±3.94 (CI −32.50, 38.40), 8.4855±3.68 (CI −24.61, 41.58), 22.84±32.13 (CI −265.844, 288.68), 6.265±6.88 (CI −55.55, 68.08), 20.945±0.67 (CI 14.90, 26.98), 20.225±11.702 (CI −84.88, 125.39), 4.2±2.27 (CI −16.25, 24.65) and 21.605±15.18 (CI −82.34, 101.01) for ATCC 90030, VPCI 226/P/20, VPCI 635/P/20, VPCI 250/P/21, VPCI 431/P/21, VPCI 944/P/21, VPCI 201/P/22 and VPCI 9b, respectively. Similarly, in the presence of glucose, the number of events showing R6G fluorescence intensity was 1.225±1.61 (CI −13.32, 15.77), 1.16±1.32 (CI −10.78, 13.10), 18.785±26.368 (CI −218.122, 255.692), 2.37±2.30 (CI −18.34, 23.08), 14.94±9.58 (CI −71.20, 101.08), 17.06±5.04 (CI −28.30, 62.42), 12.75±3.54 (CI −19.14, 44.62) and 2.215±1.61 (CI −12.33, 16.76) for ATCC 90030, VPCI 226/P/20, VPCI 635/P/20, VPCI 250/P/21, VPCI 431/P/21, VPCI 944/P/21, VPCI 201/P/22 and VPCI 9b, respectively. Panel (b) shows the graph of R6G efflux in both conditions, i.e. in the absence and presence of glucose. Every bar is plotted as the mean of the number of events showing fluorescence±sd of two independent experiments, and the CI value (both minimum and maximum) at 95% is also given. *P* value is calculated wrt ATCC 90030 which is shown above the bar. A non-significant alteration in fluorescence was seen in all strains except VPCI 9b. In the absence of glucose, the number of events showing R6G fluorescence intensity was 0.0015±0.000707 (CI −0.004, 0.007), 0.0205±0.007778 (CI −0.049, 0.09), 0.02±0.011314 (CI −0.081, 0.121), 0.0125±0.003536 (CI −0.019, 0.044), 0.095±0.018385 (CI −0.070, 0.260), 0.057±0.012728 (CI −0.05, 0.171), 0.0105±0.00495 (CI −0.033, 0.054) and 0.0305±0.00212 (CI −0.01, 0.049) for ATCC 90030, VPCI 226/P/20, VPCI 635/P/20, VPCI 250/P/21, VPCI 431/P/21, VPCI 944/P/21, VPCI 201/P/22 and VPCI 9b, respectively. Likewise, in the presence of glucose, a non-significant alteration in fluorescence was seen in all strains except VPCI 635/P/20. Here, the number of events showing R6G intensity was 0.011±0.002828 (CI −0.014, 0.036), 0.026±0.016971 (CI −0.126, 0.176), 0.0505±0.007778 (CI −0.019, 0.120), 0.028±0.015556 (CI −0.111, 0.167), 0.045±0.025456 (CI −0.183, 0.273), 0.0265±0.013435 (CI −0.094, 0.147), 0.01±0.001414 (CI −0.002, 0.022) and 0.0695±0.034648 (CI −0.241, 0.38) for ATCC 90030, VPCI 226/P/20, VPCI 635/P/20, VPCI 250/P/21, VPCI 431/P/21, VPCI 944/P/21, VPCI 201/P/22 and VPCI 9b, respectively. This experiment was done twice.

**Table 3.** Fluconazole MIC, drug resistance profile and drug efflux pump status of different strains used in the study. Expression levels are shown as fold change±sd. For the R6G assay, both influx and efflux values are given with sd. For every value of the confidence of interval (CI) at 95%, both the minimum and maximum values are given in brackets. In every case, the *P* value with ATCC 90030 is shown in bold

Strain name	Fluconazole MIC (mg l^−1^)	Resistance profile	*CDR1* expression Average±**sd** (confidence interval at 95%) *P* value wrt ATCC 90030	*CDR2* expression Average±**sd** (confidence interval at 95%) *P* value wrt ATCC 90030	*PDR1* expression Average±**sd** (confidence interval at 95%) *P* value wrt ATCC 90030	R6G influx/R6G efflux in the absence of glucose Average±**sd** (confidence interval at 95%) *P* value wrt ATCC 90030	R6G influx/R6G efflux in the presence of glucose Average±**sd** (confidence interval at 95%) *P* value wrt ATCC 90030
ATCC 90030	8.0	Susceptible	Reference	Reference	Reference	2.95±3.94, (CI −32.50, 38.40) 0.0015±0.0070 (CI −0.004, 0.007)	1.225±1.6192 (CI −0.004, 0.007) 0.011±0.002828 (CI −0.014, 0.036)
VPCI 226/P/20	64.0	Resistant	9.34684±2.012 (CI 4.35, 14.35) **0.018**	2.289907±0.6336 (CI 0.71, 3.86) **0.071**	1.028255±0.5698 (CI −0.38, 2.44) **0.939388**	8.485±3.6840 (CI −24.61,41.58) **0.02127** 0.0205±0,0113 (CI −0.049,0.09) **0.194729**	1.16±1.3293 (CI −0.049, 0.09) **0.804529** 0.026±0.01696 (CI −0.126, 0.176) **0.478056**
VPCI 635/P/20	64.0	Resistant	10.65774±0.3401 (CI 9.81, 11.50) **0.000413**	2.899312±0.7959 (CI 0.84, 4.79) **0.058**	1.041234±0.2813 (CI 0.34, 1.74) **0.823806**	22.84±31.2309 (CI −265.844,288.68) **0.500639** 0.02±0.01134 (CI −0.081, 0.121) **0.245199**	18.785±26.36801 (CI −0.081, 0.121) **0.498911** 0.0505±0.0077 (CI −0.019, 0.120) **0.056262**
VPCI 250/P/21	32.0	Susceptible dose-dependent	4.789559±0.3896 (CI 3.81, 5.75) **0.003505**	4.703971±1.123 (CI 1.91, 7.49) **0.0293**	1.235125±0.3009 (CI 0.48, 1.98) **0.308678**	6.265±6.881 (CI −55.55, 68.08) **0.356047** 0.0125±0.003536 (CI −0.019, 0.044) **0.114498**	2.37±2.3051 (CI −0.019, 0.044) **0.255073** 0.028±0.0155 (CI −0.111, 0.167) **0.415615**
VPCI 431/P/21	16.0	Susceptible	1.434845±0.2663 (CI 0.77, 2.09) **0.105625**	0.73637±0.6500 (CI −0.87, 2.35) **0.5551**	1.027561±0.8777 (CI −1.15, 3.20) **0.961571**	20.945±0.6717 (CI 14.90, 26.98) **0.081452** 0.095±0.0183 (CI −0.070, 0.260) **0.091287**	14.94±9.5883 (CI −0.070, 0.260) **0.248178** 0.045±0.02545 (CI −0.183, 0.273) **0.338506**
VPCI 944/P/21	8.0	Susceptible	2.793841±0.2611 (CI 2.14, 3.44) **0.006993**	2.319087±0.1444 (CI 1.96, 2.67) **0.003**	1.007419±0.5167 (CI −0.27, 2.29) **0.982419**	20.255±11.7026 (CI −84.88, 125.39) **0.36217** 0.057±0.0127 (CI −0.05, 0.171) **0.107925**	17.06±5.0487 (CI −0.05, 0.171) **0.184237** 0.0265±0.01343 (CI −0.094, 0.147) **0.2869**
VPCI 201/P/22	64.0	Resistant	1.376057±0.2458 (CI 0.76, 1.99) **0.117817**	1.21697±0.5095 (CI −0.053, 2.47) **0.544828**	0.619856±0.2121 (CI 0.09, 1.14) **0.089987**	4.2±2.2768 (CI −16.25, 24.65) **0.4816** 0.0105±0.00495 (CI −0.33, 0.054) 0.204833	12.75±3.5496 (CI −033, 0.054) **0.1995808** 0.01±0.0014 (CI −0.002, 0.022) **0.5**
VPCI 9b	64.0	Resistant	12.80769±1.5352 (CI 8.99, 16.6) **0.005588**	1.388657±0.2524 (CI −0.76, 2.01) **0.116585**	1.464108±0.4671 (CI 0.28, 2.64) **0.233416**	21.605±15.1815 (CI −82.34, 101.01) **0.256319** 0.0305±0.0021 (CI −0.01, 0.049) **0.043835**	2.215±1.6192 (CI −0.01, 0.049) **9.58E−09** 0.0695±0.03464 (CI −0.2418, 0.38) **0.270779**

In VPCI 9b, less accumulation and more efflux of R6G was seen in the presence of glucose in comparison to the absence of glucose, similar to VPCI 226/P/20 and VPCI 635/P/20, respectively. In fact, like RT PCR, maximum drug efflux was seen in VPCI 9b only (Figs24a, b and Table 3).ANOVA results for significance testing of this experiment are shown in Table 2.

### *PDR1* sequencing

GOF mutation in *PDR1* plays a significant role in *CDR1* overexpression and drug efflux in *C. glabrata;* therefore, *PDR1* sequencing was done for all eight strains used in the study and was compared to the reference strain CBS138/ATCC 2001 (gene ID: 2886430). On sequencing, it was seen that although many mutations were seen in central regulatory domain (CRD) and transcriptional activation domain of *PDR1* ranging from amino acid position 254–969 and 969–1,107, respectively, L328F was one novel GOF mutation which was found in all resistant strains except VPCI 201/P/22 (Table 4).

**Table 4.** Mutations in *PDR1*, *MSH2* and *ERG11* in different strains used in the study, along with their MIC_50_ and drug profile for fluconazole. Mutations shown with an asterisk symbol are found in hot spot region 1 of *ERG11*

Strain name	Fluconazole MIC (mg l^−1^)	Drug profile	Mutations seen in *PDR1*	Mutations seen in *MSH2*	Mutations seen in *ERG11*
ATCC 90030	8.0	Susceptible	Wild type	P208S	V36A, Q47H, L52W, R53K, L59F, R56K
VPCI 226/P/20	64.0	Resistant	L328F, R401Q, C914S, A918E,	P208S	Q47H, L52W, E340D, Y342F, Q343L, D381F
VPCI 635/P/20	64.0	Resistant	L328F, L924Q	V239L	Q47H, Q85K, I91F
VPCI 250/P/21	32.0	Susceptible dose-dependent	R295G, D1035G, T1074A, L1078F, D1082E, V1085A, F1102L, S1103Y	No mutation	Y42S, Q47H, R53K, R56K, Q369H, M370I
VPCI 431/P/21	16.0	Susceptible	L669I	No mutation	Y42S, T43P, Q47H, L52K, R53K, R56K, W66D, I91L, L97W, R99K,
VPCI 944/P/21	8.0	Susceptible	No mutation seen	No mutation	V36A, L52W, Q85K, I91L, R56K
VPCI 201/P/22	64.0	Resistant	P258S, T829P, V835G, A918E, I938N	V239L	T43P, R53K, Q85K, K235N, G236F
VPCI 9b	64.0	Resistant	L328F, V835G	V239L	V41A, T43P, Q47H, Y50S, Q85K, I91L, L97W E111D*, E117K*, R403T

This novel mutation L328F was also found in VPCI 9b but not in its parental strain VPCI 431/P/21 (Table 4) . *PDR1* sequences of all strains used in the study are added to the GenBank database, and their accession numbers are given in Table 5.

**Table 5.** GenBank accession numbers of *PDR1*, *MSH2* and *ERG11* of different strains used in the study

Strain name	GenBank accession no. of *PDR1*	GenBank accession no. of *MSH2*	GenBank accession no. of *ERG11*
ATCC 90030	PV269801	PV364181	PV269793
VPCI 226/P/20	PV269802	PV364182	PV269794
VPCI 635/P/20	PV269803	PV364183	PV269795
VPCI 250/P/21	PV269804	PV364184	PV269796
VPCI 431/P/21	PV269805	PV364185	PV269797
VPCI 944/P/21	PV269806	PV364186	PV269798
VPCI 201/P/22	PV269807	PV364187	PV269799
VPCI 9b	PV269808	PV364188	PV269800

### Correlation between fluconazole susceptibility and *MSH2* genotypes

#### *MSH2* sequencing

*MSH2* genotype is a known hallmark for fluconazole resistance in Indian isolates of *C. glabrata* [34]; therefore, *MSH2* sequencing was done for all strains used in the study, and they were compared to the reference strain CBS138/ATCC 2001 (gene ID: 2889005). We found that P208S was one non-synonymous mutation that was found in ATCC 90030 and VPCI 226/P/20 only, while V239L was another known mutation found in VPCI 635/P/20 and VPCI 201/P/22, respectively (Table 4).

This non-synonymous mutation, V239L, was also found in VPCI 9b but not in its parental strain, VPCI 431/P/21 (Table 4). *MSH2* sequences of all strains used are added to the GenBank database, and their accession numbers are given in Table 5.

### The ergosterol biosynthesis pathway is not compromised in *C. glabrata*

#### *ERG11* expression is not altered in *C. glabrata*

**Fig. 5.**
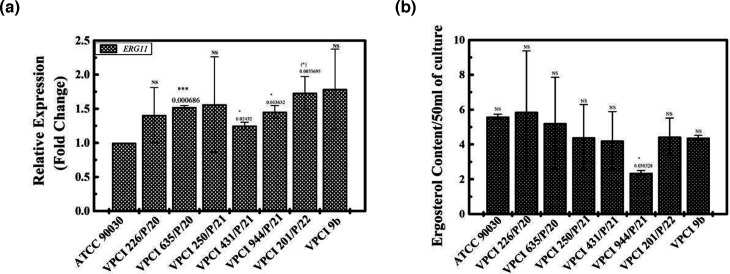
Effect on *ERG11* expression and ergosterol content in different strains of *C. glabrata*: Panel (a) shows the transcript levels of *ERG11* in different strains of *C. glabrata* using ATCC 90030 as a control. Transcript levels are shown as fold change, which is shown as mean±sd of three independent experiments. The CI value (both minimum and maximum) at 95% is also given. *P* value is calculated wrt ATCC 90030, which is shown above the bar. In comparison to ATCC 90030, transcript levels were upregulated to 1.407±0.40 (CI, 0.40, 2.41), 1.524±0.023 (CI 1.46, 1.58), 1.563±0.700 (CI −0.176, 3.30), 1.25±0.050 (CI 1.12, 1.36), 1.45±0.093 (CI 1.22, 1.68), 1.73±0.23 (CI 1.13, 2.32) and 1.78±0.58 (CI 0.33, 3.24) in VPCI 226/P/20, VPCI 635/P/20, VPCI 250/P/21, VPCI 431/P/21, VPCI 944/P/21, VPCI 201/P/22 and VPCI 9b, respectively. This experiment was done thrice. Panel (b) shows the ergosterol content of different strains of *C. glabrata* using ATCC 90030 as a control. Sterol content was plotted as grams per 50 ml of culture, and no major difference in ergosterol content was seen. Data are shown as mean±sd of two independent experiments. The CI value (both minimum and maximum) at 95% is also given. *P* value is calculated wrt ATCC 90030 which is shown above the bar. In ATCC 90030, ergosterol levels were found to be 5.6±0.141 g/50 ml (CI 4.32, 6.87) in VPCI 226/P/20, 5.87±3.50 g/50 ml (CI −25.64, 37.38) in VPCI 635/P/20, 5.23±2.53 g/50 ml (CI −18.40, 28.86) in VPCI 250/P/21, 4.41±1.88 g/50 ml (CI −12.48, 21.30) in VPCI 431/P/21, 4.22±1.66 g/50 ml (CI −10.70, 19.15) in VPCI 944/P/21, 2.36±0.13 g/50 ml (CI 1.15, 3.57) in VPCI 201/P/22, 4.45±1.06 g/50 ml (CI −5.07, 13.98) and 4.39±0.12 g/50 ml (CI 3.24, 5.53) in VPCI 9b, respectively. This experiment was done twice.

The ergosterol biosynthesis pathway was also studied, though it does not play any role in azole resistance in *C. glabrata.* Here, first *ERG11* expression was studied by quantifying the transcript levels, and we found that compared to ATCC 90030 no major overexpression of *ERG11* was seen in any clinical strain, and all were showing the same expression irrespective of fluconazole MIC, except VPCI 201/P/22, where a little overexpression was seen (Fig. 5a and Table 6).

**Table 6.** Fluconazole, MIC, drug resistance profile and drug efflux pump status of different strains used in the study*. ERG11* expression levels are written as fold change±sd. Ergosterol content is shown as grams per 50 ml. For every value of the confidence interval at 95%, both the minimum and maximum values are given in brackets. In every case, the *P* value with ATCC 90030 is shown in bold

Strain name	Fluconazole MIC (mg l^−1^)	Resistance profile	*ERG11 e*xpression (fold change) Average±**sd** (confidence interval at 95%) *P* value wrt ATCC 90030	Ergosterol content (grams/50 ml) Average±**sd** (confidence interval at 95%) *P* value wrt ATCC 90030
ATCC 90030	8.0	Susceptible	Reference	5.87±0.1414 (CI 4.32, 6.87)
VPCI 226/P/20	64.0	Resistant	1.407812±0.4049 (CI 0.40, 2.41) **0.223231**	5.87±3.50725 (CI −25.64, 37.38) **0.928086**
VPCI 635/P/20	64.0	Resistant	1.524621±0.02380 (CI 1.46, 1.58) **0.000686**	5.23±2.6304 (CI −18.884, 28.86) **0.868086**
VPCI 250/P/21	32.0	Susceptible dose-dependent	1.563977±0.7000 (CI −0.176, 3.30) **0.29797**	4.41±1.8809 (CI −12.48, 21.30) **0.510522**
VPCI 431/P/21	16.0	Susceptible	1.24811±0.0478 (CI 1.12, 1.36) **0.02432**	4.225±1.6617 (CI −10.70, 19.15) **0.422433**
VPCI 944/P/21	8.0	Susceptible	1.45596±0.0931 (CI 1.22, 1.68) **0.013632**	2.365±0.1343 (CI 1.15, 3.57) **0.03832**
VPCI 201/P/22	64.0	Resistant	1.733897±0.2349 (CI 1.13, 2.32) **0.033695**	4.45±1.0606 (CI −5.07, 13.98) **0.32751**
VPCI 9b	64.0	Resistant	1.788248±0.5864 (CI 0.33, 3.24) **0.14533**	4.39±0.1272 (CI 3.24, 5.53) **0.099955**

*ERG11* overexpression was also seen in VPCI 9b compared to both ATCC 90030 and VPCI 431/P/21, respectively (Fig. 5a and Table 6).

Descriptive statistics are shown in Table 2.

#### Ergosterol levels are not altered in *C. glabrata*

Ergosterol levels were also measured in all strains by GCMS where absolute quantification of ergosterol was done and is shown as grams per 50 ml on the *Y*-axis in the graph (Fig. 5b). It was found that compared to ATCC 90030, no major difference in ergosterol content was seen in any clinical strain, irrespective of fluconazole MIC. All strains were showing almost the same ergosterol content except VPCI 944/P/21 (MIC 8.0 mg l^−1^), where a decrease in ergosterol content was seen (Fig. 5b and Table 6).

In VPCI 9b, no difference in ergosterol content was seen, and it was almost the same as ATCC 90030 and VPCI 431/P/21, respectively (Fig. 5b and Table 6).

Descriptive statistics are shown in Table 2, which correlated with the results.

### *ERG11* sequence analysis

On sequencing *ERG11* compared to the reference genome CBS 138/ATCC 2001 (gene ID: 2887532), although many non-synonymous mutations were seen, they were not significant in azole resistance (Table 4).

Similarly, on sequencing *ERG11* in VPCI 9b, like other strains, many non-synonymous mutations were seen, but mutations like V41A, Y50S, E111D and K117E were exclusively found in VPCI 9b. Notably, mutations found at position 111 (E111D) and 117 (K117E) were located in hot spot region 1 (HS1) of *ERG11,* respectively (Table 4) [55]. *ERG11* sequences of all strains used are added to the GenBank database, and their accession numbers are given in Table 5.

### Biofilm assay

#### Biofilm quantification

Biofilm quantification was done using the XTT–menadione method both in the presence and absence of serum, where it was found that out of all strains, including VPCI 9b, only VPCI 201/P/22 showed biofilm formation both in the absence and presence of serum (Fig. S3A). On ANOVA testing, a significant difference was seen both in the absence and presence of serum, concluding that the results seen were not random but were due to variations in strains. Statistics are shown in Table 2.

#### Morphological observation of biofilm

Morphological study of biofilm was done by taking images of all strains both in the absence and presence of serum by SEM, but no major difference was seen except VPCI 201/P/22, where a little difference was seen Fig. S3B.

### Transmission electron microscopy

Transmission electron microscopy (TEM) was done to see the anatomical effect of drug resistance on the cell wall, but due to cost constraints, this study was done for VPCI 635/P20 and VPCI 9b only, along with their respective controls ATCC 90030 and VPCI 431/P/21. Here, no major anatomical difference was seen in any strain except for some black dots called chitosans seen on the cell walls of resistant strains. These chitosan levels were found in large amounts in VPCI 9b, which differentiated this resistant strain from VPCI 635/P/20. Results are shown in Fig. S4.

## Discussion

This study reports the isolation of fluconazole-resistant clinical isolates of *C. glabrata* from India along with their resistance mechanism, and to the best of my knowledge, this is the first study from India that reports the resistance mechanism in *C. glabrata*. This study was conducted on 240 clinical isolates in a span of 5 years, where only 3 fluconazole-resistant isolates were found, which accounts for 1.25% which is much less than that seen in Western countries where 10–50% of fluconazole resistance is seen. In Australia, it is 6.8%, in Saudi Arabia, it is 50%, while in China, it ranges from 6.2%−20.3% [5,18, 56, 57]. Even though there are studies from India that report higher resistance, they do not show the resistance mechanism,[35,58] and therefore, in this study, we are studying and comparing the resistance mechanism in clinical vs. induced fluconazole-resistant strains.

There are various pathways that play a role in drug resistance with different mechanisms, and therefore, to understand the reason behind this resistance, first, drug efflux pumps (Cdr1p and Cdr2p) were studied since these are the major factors responsible for drug resistance in *C. glabrata* [24,59]. Overexpression of these drug pumps leads to drug resistance by effluxing more drug out of the cell, which was studied by the R6G assay by monitoring both its influx and efflux. Here, it was seen that in all resistant strains, only *CDR1* was overexpressed, while *CDR2* was not showing any overexpression or very little overexpression compared to *CDR1,* thus demonstrating that it is not involved in drug resistance, as seen in previous studies [24,60]. Pdr1, which is a transcription factor for drug efflux pumps, correlates with Cdr1p and regulates its efflux activity [26,61]. It is mostly upregulated in resistant isolates, but here in this study, its expression was not altered; rather, it was almost the same in all strains, irrespective of drug susceptibility. On sequencing, one novel GOF mutation, L328F, was seen in the CRD region of *PDR1* in all resistant strains except VPCI 201/P/22, where neither *CDR1* overexpression nor any GOF mutation was seen. This strain was also not showing any effect on R6G activity, concluding that in this particular strain, the Cdr1-Pdr1 circuit was not active and efflux pumps were not involved in drug resistance.

We then looked for other pathways responsible for drug resistance, and for that, we first monitored *MSH2,* which is one of the important genes of the mismatch repair pathway and shows some important mutations like V239L in resistant isolates. Although this mutation is not universal, it is most commonly seen in Asian and Southeast Asian countries [18,62, 63].

Next, we studied the ergosterol biosynthesis pathway, which is an important pathway in drug resistance in *Candida* spp. but in *C. glabrata,* it is not known to play any significant role in drug resistance [64,65]. In this study, we also did not find any major difference in *ERG11* expression (except VPCI 201/P/22 and VPCI 9b) or in ergosterol levels. On sequencing, no GOF mutation was seen in any resistant strain which was responsible for this phenotype. But interestingly, two GOF mutations were seen in the hot spot region (HS1) of *ERG11* in VPCI 9b, which was showing the highest *ERG11* overexpression among all resistant strains and was probably responsible for the resistant phenotypes.

Biofilm formation is another factor responsible for drug resistance and induces resistance by forming a shield of cells of different phenotypes which does not allow antifungal drugs to reach the target cell and hence resistance is seen. There are different phenotypes like yeast cells, pseudo-hyphae and hyphae seen in biofilm formation. Hyphae formation is mainly seen in biofilm in *C. albicans* and other *Candida* spp*.,* but in *C. glabrata*, only yeast cells are seen [66,67]. To study this, an *in vitro* biofilm study was done both in the absence and presence of serum to mimic the human body environment and to see its effect on host cells, but no major difference was seen in any strain except VPCI 201/P/22, where a significant difference in biofilm formation was seen both in the absence and presence of serum. Although this strain was showing resistant phenotypes, as discussed above, the role of Cdr1p and not even Erg11p was seen, and hence, it was concluded that in this particular strain, biofilm formation is the major reason for azole resistance. But since this difference was seen in just one strain, it can be the preliminary result and needs to be investigated further with more clinical isolates as per Indian standards. TEM was also done to find the anatomical alterations taking place in the cell in the presence of different susceptibilities of the drug, and as seen in literature, different colloidal particles called chitosans were found on the cell wall of resistant isolates, probably to overcome the cellular stress generated due to a high amount of drug [68].

**Fig. 6.**
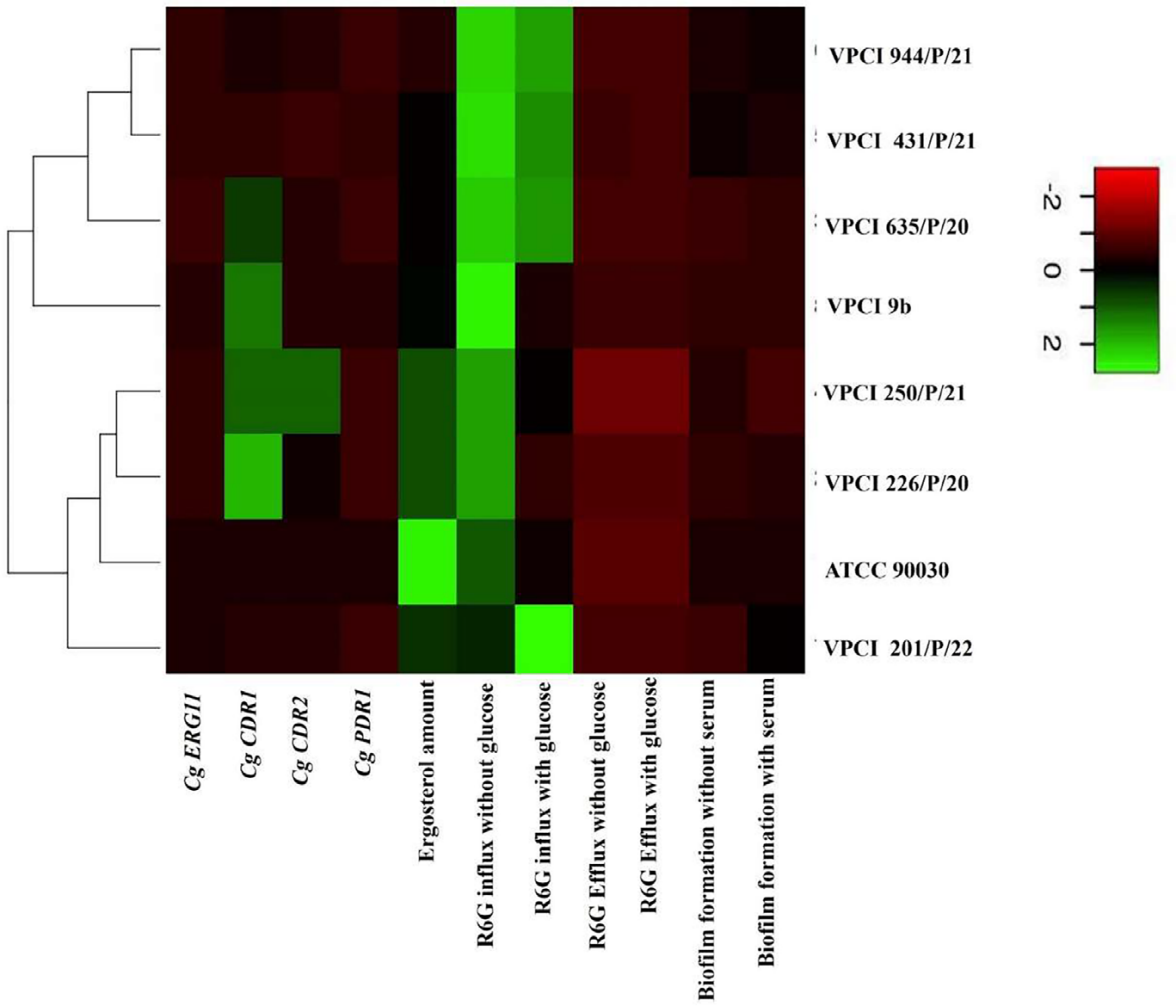
Heat map showing difference between resistance profiles of different strains of *C. glabrata* used in study: Heat map is made to show the difference between the resistance profiles of different strains of *C. glabrata* used in the study. This is an integrated figure that shows all expressions in a single frame. The differentiating value ranges from −2 to 2 based on which colour is chosen. Here, *CDR1* expression and R6G accumulation are the major contributing factors in deciding the resistance profile.

**Fig. 7.**
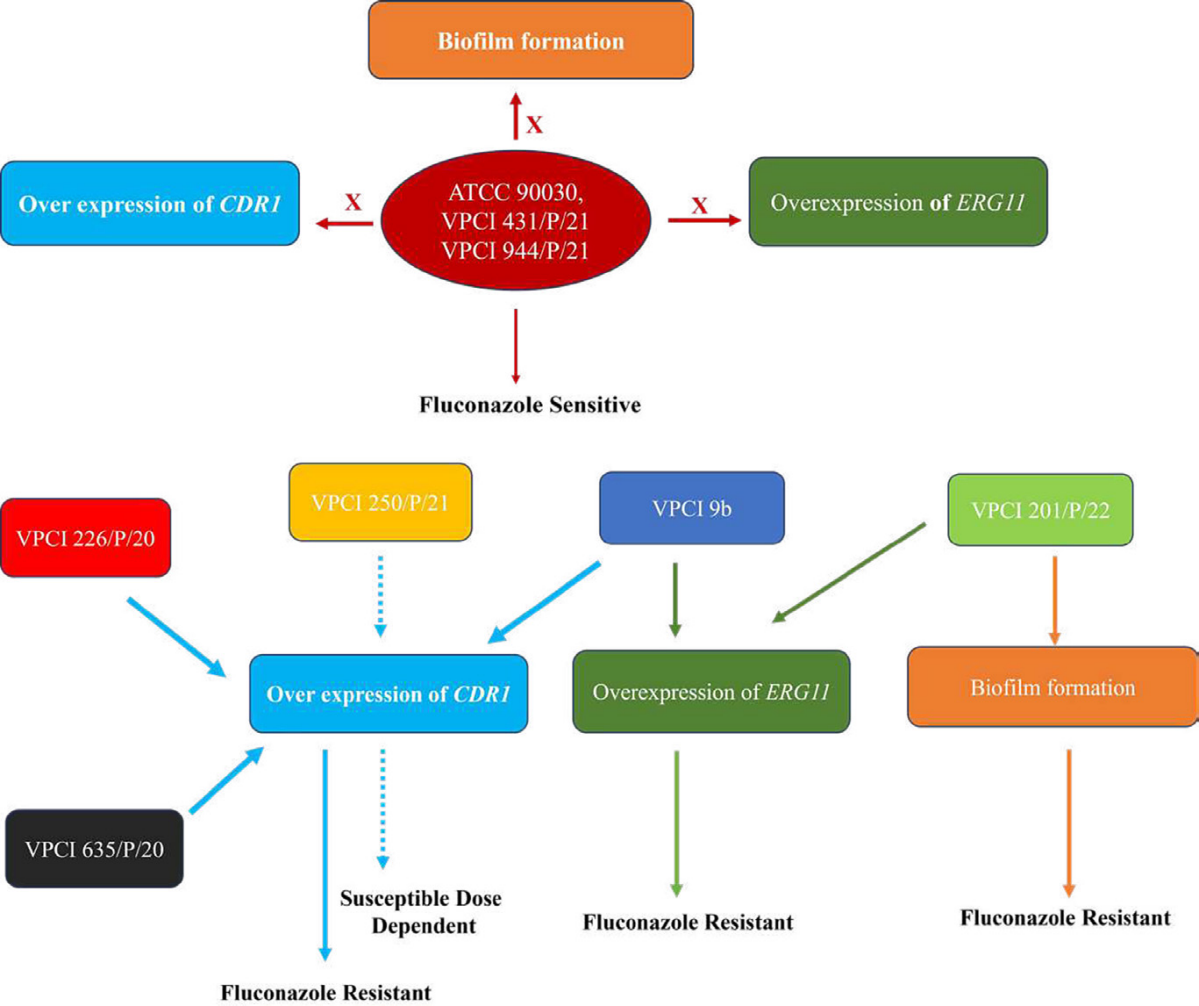
Visual model showing the resistance profiles of different strains of *C. glabrata* used in the study: a visual map is made to show the difference between the resistance profiles of different strains of *C. glabrata* used in the study. This figure shows which pathway is affected in which strain and contributes to resistance. Here, bold lines indicate the resistance profile, while a dotted line indicates the SDD profile.

Based on all these phenotypes, one heat map (Fig. 6) and one visual model (Fig. 7) were made showing characteristics of all the strains collectively in one place, where differentiation was done based on colour intensity, while the visual model showed the resistance profiles of different strains and how they were interconnected. These figures were helpful in analysing the complete data in a single view. In addition, (Table 7) shows the types of experiments done against different strains, making the study more understandable.

**Table 7.** Table showing complete list of types of experiments done for different strains in this study

	ATCC90030	VPCI 226/P/20	VPCI 635/P/20	VPCI 250/P/21	VPCI 431/P/21	VPCI 944/P/21	VPCI 201/P/22	VPCI 9b
Spot assay	√	√	√	√	√	√	√	√
E-test	√	√	√	√	√	√	√	√
Target sequencing (*ERG11*/*MSH2*/*PDR1*)	√	√	√	√	√	√	√	√
Sterol estimation	√	√	√	√	√	√	√	√
Rhodamine assay	√	√	√	√	√	√	√	√
Real-time PCR (*CDR1*, *CDR2 PDR1* and *ERG11*)	√	√	√	√	√	√	√	√
Biofilm	√	√	√	√	√	√	√	√
SEM	√	√	√	√	√	√	√	√
TEM	√		√		√			√

## Conclusion

In this study, we are showing fluconazole resistance in Indian clinical isolates of *C. glabrata* where resistance pathways were also studied. Here, we found a significant increase in the expression level of *CDR1* of drug efflux pumps belonging to the ATP-binding cassette superfamily, and a novel GOF mutation in *PDR1* controlling this circuit was also seen. *ERG11* expressions were not altered, but interestingly, little overexpression was seen in one clinical strain and one synthesized strain, and notably, mutations were also seen in the HS1 region of *ERG11* of the synthesized strain. Biofilm formation was also found to be responsible for azole resistance as seen in one of the strains used in this study, and all these factors contribute to the knowledge of the scientific and clinical community. This finding can be taken as a reference which can underscore the complexity of resistance mechanisms in *C. glabrata* and pave the way for further research into innovative therapeutic strategies.

## Limitations of the study

Although this study can contribute significantly to the scientific and clinical community, this study has certain limitations and one important limitation was the sample size. The sample size used in this study was very small because out of a total of 240 isolates collected, we were able to find only three fluconazole-resistant strains and one SDD strain, and for comparing the resistance mechanism, all phenotypes (susceptible, SDD and resistant) were needed. Therefore, we randomly collected two susceptible isolates from the collection and used them in the study. Another limitation of the study was that various omics studies like whole-genome sequencing or transcriptomic studies by RNA-seq should have been done for a better understanding of the result, but due to cost constraints and time limitations, these studies could not be done. However, in the future, these approaches can be used for understanding the resistance mechanism. The third limitation of the study, which we found, was that, as per the results seen, biofilm formation was also found to be one of the factors responsible for drug resistance. However, since this result was seen in a single strain only and lacks a wider approach, this study can be used as a preliminary study and should have been done in a larger number of samples.

## Supplementary material

10.1099/jmm.0.002083 Uncited Fig. S1.

## Abbreviations

AFG: anidulafungin
AFST: antifungal susceptibility testing
AMB: amphotericin B
BMD: broth microdilution
CFG: caspofungin
CLSI: Clinical and Laboratory Standards Institute
CRD: central regulatory domain
5-FC: 5-flucytosine
FLU: fluconazole
GM: geometric mean
GOF: gain-of-function
ISA: isavuconazole
ITC: itraconazole
MFG: micafungin
NAC: non-albicans *Candida*
POS: posaconazole
R6G: rhodamine 6G
SDD: susceptible-dose dependent
SEM: scanning electron microscopy
TEM: transmission electron microscopy
VRC: voriconazole
