# Supplementary material for: Mechanism of azole resistance in Candida glabrata isolates from India: clinical vs. induced perspectives
Source: J Med Microbiol. 2025 Nov 25;74(11):002083. doi: 10.1099/jmm.0.002083 (PMC12646537; doi:10.1099/jmm.0.002083)
Supplement: Uncited Fig. S1. [file jmm-74-02083-s001.pdf]

Mechanism of azole resistance in *Candida glabrata* isolates from India: Clinical vs Induced perspectives

SUPPLEMENTARY DATA

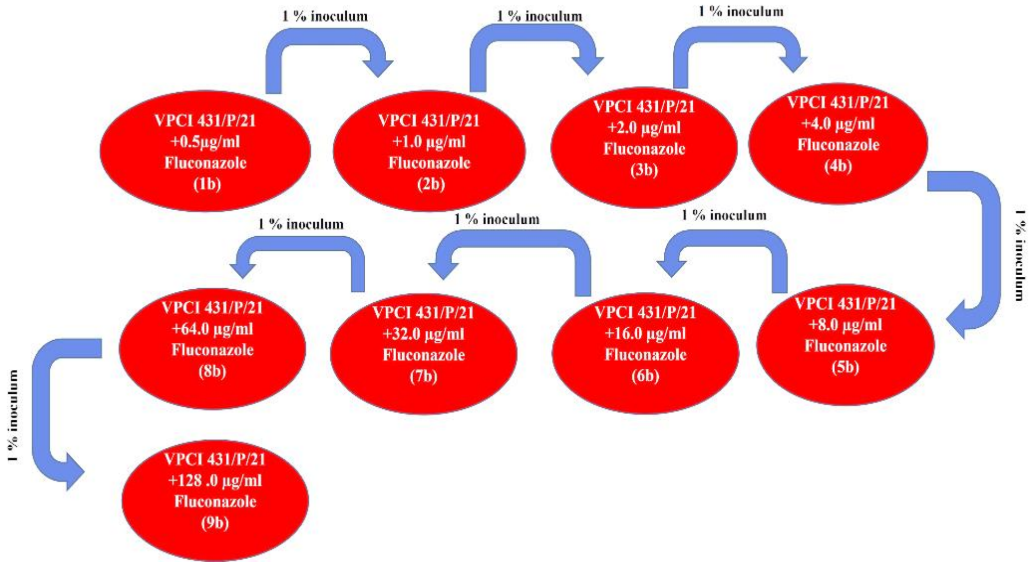

Supplementary Fig. 1. In vitro synthesis of a fluconazole-resistant strain of *Candida glabrata*: The figure illustrates the stepwise induction of fluconazole resistance in the laboratory, where the parental strain VPCI 431/P/21 was sequentially transferred in gradually increasing concentrations of fluconazole (0.5 µg/ml to 128 µg/ml). At each step, 1% inoculum from the previous concentration was transferred to the next higher concentration and cultured overnight. Samples from each transfer were collected, and the minimum inhibitory concentration (MIC) was determined. The process ultimately resulted in the generation of the fluconazole-resistant derivative strain, VPCI 9b.

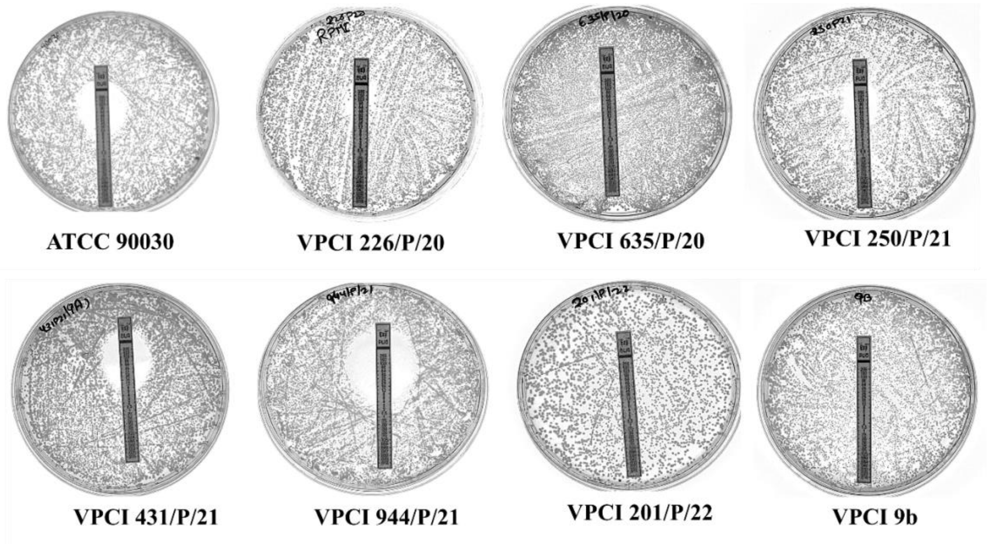

Supplementary Fig. 2. Effect of increasing concentrations of fluconazole on clinical and laboratory-generated strains of *Candida glabrata*: The figure shows the activity of fluconazole E-test strips (Hi-Media) against different *C. glabrata* strains. Cell suspensions (100 µL, adjusted to OD_{600} = 0.1) were uniformly spread on RPMI agar plates, and E-test strips were placed in the centre. Plates were incubated at 37 °C for 24 h, and representative images were captured. Each experiment was performed twice.

A

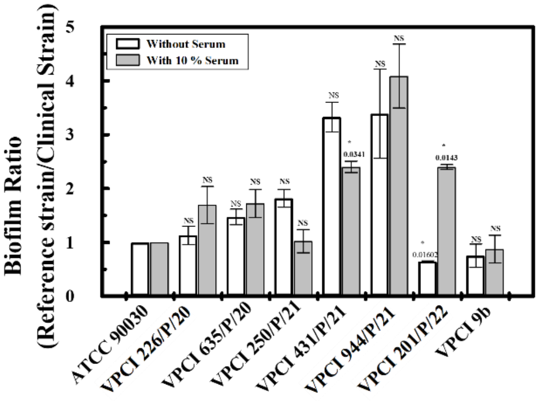

B

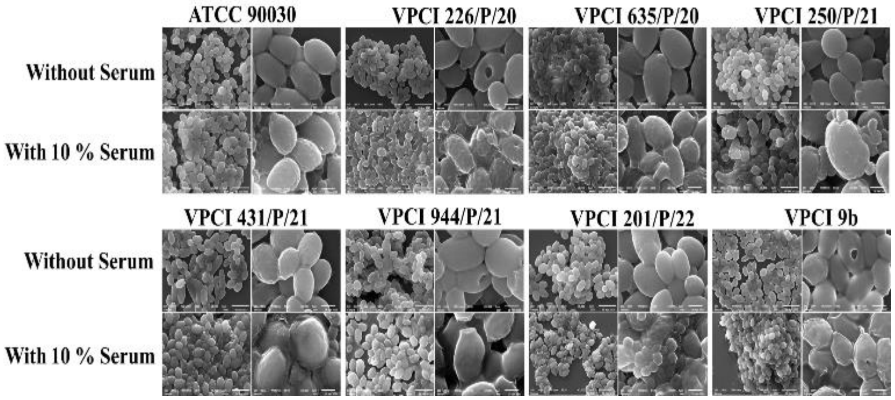

Supplementary Fig. 3. Effect of serum on biofilm formation in reference and clinical strains of *Candida glabrata*. **Panel A.** Quantification of biofilm formation in the absence and presence of 10% serum. Biofilm biomass was measured using the XTT reduction assay and expressed as the biofilm ratio of each clinical strain relative to the reference strain ATCC 90030. Data represent mean ± SD of three independent experiments, along with 95% confidence intervals (minimum and maximum). P values are shown above the bars relative to ATCC 90030. In the absence of serum, biofilm ratios compared to ATCC 90030 were: VPCI 226/P/20 (1.13 ± 0.17, CI 0.39–2.67), VPCI 635/P/20 (1.47 ± 0.14, CI 0.17–2.70), VPCI 250/P/21 (1.82 ± 0.17, CI 0.32–3.31), VPCI 431/P/21 (3.33 ± 0.28, CI 0.84–5.80), VPCI 944/P/21 (3.39 ± 0.83, CI 4.03–10.82), VPCI 201/P/22 (0.65 ± 0.01, CI 0.53–0.75), and VPCI 9b (0.75 ± 0.22, CI 1.18–2.69). In the presence of serum, biofilm ratios were: VPCI 226/P/20 (1.69 ± 0.34, CI 1.38–4.77), VPCI 635/P/20 (1.72 ± 0.26, CI 0.62–4.07), VPCI 250/P/21 (1.02 ± 0.22, CI 0.92–2.96), VPCI 431/P/21 (2.40 ± 0.11, CI 1.44–3.35), VPCI 944/P/21 (4.09 ± 0.59, CI 1.25–9.42), VPCI 201/P/22 (2.40 ± 0.44, CI 1.99–2.79), and VPCI 9b (0.87 ± 0.26, CI 1.46–3.21) **Panel B**. Visualization of biofilm architecture in the absence and presence of 10% serum. Cells (OD_{600} = 0.5) were incubated for 48 h in 24-well flat-bottom plates, washed, fixed, and dehydrated with graded ethanol (as described in Methods). Biofilm structures were examined using scanning electron microscopy (JEOL) at 30 kV and magnifications ranging from 5000× to 25,000×. This experiment was repeated twice.

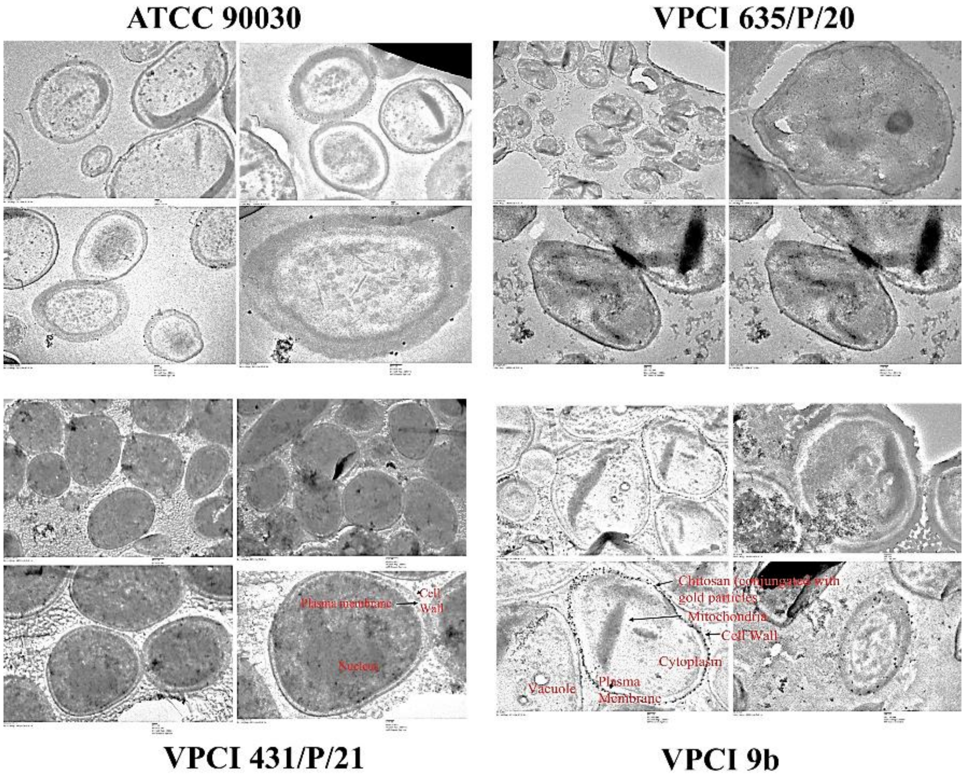

Supplementary Fig. 4. Azole resistance is associated with cell wall and ultrastructural alterations in *Candida glabrata*. Transmission electron microscopy (TEM) images of four strains—ATCC 90030 (reference strain), VPCI 431/P/21 (clinical fluconazole-susceptible strain), VPCI 635/P/20 (clinical fluconazole-resistant strain), and VPCI 9b (laboratory-generated resistant strain) were analyzed. Resistant strains showed distinct alterations in the cell wall and overall cellular architecture. Additionally, accumulation of gold-coated chitosan particles was observed in resistant strains. The experiment was performed once.

Supplementary Table 1: List of primers used in study

Cgla_ERG11 FP (1)	ATAATGGGCGATCCCTTCATGT
Cgla_ERG11 FP (2)	ACCAAGCCATACGAGTTCTTC
Cgla_ERG11 FP (3)	AGGTAAGGAAATGAGAGAC
Cgla _ERG11 FP (4)	AGGAATTGACTTACGATGAC
Cgla_ERG11 RP	AATCAGCCGTATATCCCGTATA
Cgla_MSH2 FP (1)	AACATCAGATAAACTGTCTAAAG
Cgla_MSH2 FP *(2*	ATGTTATATTGGGGTTGACA
Cgla_MSH2 FP (3)	GGTTAAAGCAACCTTTGACA

Cgla _MSH2 FP (4)	ATATTGAGCTCTCAACGGTT
Cgla_MSH2 RP	TCGAGGCACTCAGTCAGTAACAA
Cgla_PDR1 FP (1)	CCCCATATCGTATTGCCATTG
Cgla _PDR1 FP (2)	TTGTGGGTAATCCACTAGC
Cgla_PDR1 FP (3)	CTAACACAGGCAGTTAATG
Cgla _PDR1 FP (4)	GAGTCGATATCGATTGCCA
Cgla _PDR1 FP (5)	ACGATTCCATTGTCACAGA
Cgla _PDR1 FP (6)	GGCCCTTACAATAGTTACCA
Cgla _PDR1 FP (7)	AGATACAGCAGCAGCTAGC
Cgla _PDR1 FP (8)	TGCCCTGACCTTGCAGATC
Cgla _PDR1 RP	CGCTAATTTGAGGTAGTCTA
Cgla_ACT11 RTF	TTTGGACTCTGGTGACGGTG
Cgla_ACT11 RTR	ACCAGCAAGGTCGATTCTCAA
*Cgla _CDR1*_RTF	GGTGCCATCCACAGCCTCTT
*Cgla _CDR1*_RTR	GGCGATGTTGAAACCGTGGG
*Cgla _CDR2*_RTF	ACGACTACGATGCCCGTCTG
*Cgla _CDR2*_RTR	CCCGATCGAGTACGGCTTGT
*Cgla _PDR1*_RTF	ACTGCATCTCCCTTATCGGGC
*Cgla _PDR1*_RTR	AGTGGGCACGTCAGAGACAG
*Cgla _ERG11*_RTF	GGTCGCCTTGCCATTTGTGT
*Cgla _ERG11*_RTR	GGATAGCAGAGCCGACCCAT
